# Improving Nutritional and Health Benefits of Biscuits by Optimizing Formulations Based on Sprouted Pseudocereal Grains

**DOI:** 10.3390/foods11111533

**Published:** 2022-05-24

**Authors:** Luz María Paucar-Menacho, Wilson Daniel Simpalo-López, Williams Esteward Castillo-Martínez, Lourdes Jossefyne Esquivel-Paredes, Cristina Martínez-Villaluenga

**Affiliations:** 1 Departamento de Agroindustria y Agronomía, Facultad de Ingeniería, Universidad Nacional del Santa, Chimbote 02711, Peru; luzpaucar@uns.edu.pe (L.M.P.-M.); wsimpalol@uns.edu.pe (W.D.S.-L.); wcastillo@uns.edu.pe (W.E.C.-M.); lourdes.ep@gmail.com (L.J.E.-P.); 2 Department of Technological Processes and Biotechnology, Institute of Food Science, Technology and Nutrition (ICTAN-CSIC), 28040 Madrid, Spain

**Keywords:** bioactive compounds, biscuits, digestion, formulation, pseudocereals, sprouting

## Abstract

A mixture design (MD) was used to evaluate the effect of replacing wheat flour (WF) with sprouted cañihua (*Chenopodium pallidicaule* Aellen), kiwicha (*Amarathus caudatus* L.), and quinoa (*Chenopodium quinoa* Willd.) flours (SCF, SKF, and SQF, respectively) on the content of phytic acid (PA), γ-aminobutyric acid (GABA), total soluble phenolic compounds (TSPC), and antioxidant activity (AA) in biscuits. Generally, sprouted pseudocereal flours contained lower amounts of starch and protein, comparable fat, ash, PA content, and increased levels of bioactive compounds (GABA and TSPC) and AA compared with wholegrain flours. Moreover, it was confirmed that sprouted pseudocereal flours were nutritionally superior to refined WF. MD allowed the modeling of target parameters showing that PA, GABA, TSPC, and AA were positively influenced by the proportion of flours in the biscuit. The models that better described the variation in nutritional parameters as a function of the formulation displayed typically linear and binary interactions terms. SKF exerted the highest influence on the increased content of PA. Therefore, to increase mineral bioavailability, the use of SCF and SQF in the formulation of biscuits was suggested. SCF and SQF positively influenced in GABA, TSPC, and AA in biscuits. The optimal ternary blends of flours that maximize the content of bioactive compounds and AA of biscuits and simultaneously minimize PA content were identified. To study the fate of biscuits in digestion, the optimal formulation for biscuits containing SQF/SCF was selected. For this type of baked product, reduced starch digestibility and glycemic index was observed compared with the control (100% WF). Moreover, the amounts of bioaccessible GABA, TSPC, and AA were higher in gastric and intestinal digests compared with control biscuit. Overall, these results highlighted the nutritional and health benefits of incorporation of flours from sprouted Andean grains in the production of biscuits.

## 1. Introduction

The 2030 Agenda of the United Nations for Sustainable Development pointed out the need to develop sustainable food systems to deliver healthy diets for a growing population [1]. To achieve this global objective, it is necessary to give priority to crops with improved efficiency in terms of the use of natural resources and better nutritional value to meet the requirements for a healthy diet. Within this framework, pseudocereal crops have received much attention as contributors to food security as their cultivation is possible in regions with a harsh climate and poor soil conditions. Pseudocereals grains are edible seeds belonging to dicotyledonous species that are known as such due to their similar physical appearance and high starch content, similar to true cereals. Two of the most common pseudocereals belong to the *Amaranthus* and *Chenopodium* genera. The former includes nearly 60 species, but only three are cultivated for grain production (*Amaranthus hypochondriacus*, *Amaranthus caudatus*, and *Amaranthus cruentus*). Cañihua (*Chenopodium pallidicaule* Aellen) and quinoa (*Chenopodium quinoa* Willd.) are two cultivated species within the *Chenopodium* genus. Pseudocereal grains are a good source of protein with a well-balanced amino acid profile, unsaturated fatty acids, dietary fiber, and minerals [1,2]. Besides being highly nutritious, these pseudocereal crops are considered to be rich in health-promoting bioactive compounds (phenolic compounds, phytoecdysteroids, carotenoids, etc.) [1]. However, the presence of antinutrients in pseudocereals (phytic acid [PA], saponins, tannins, and protease inhibitors) may restrict their application in food industry [2,3].

The popularity of grain germination is re-emerging in the development of healthy foods. The launch of products with sprouted grains has increased exponentially since 2006 [4,5]. Sprouted grains have found application as ingredients in several food product categories because of their high nutritional value, interesting sensory attributes, and technological properties. In particular, sprouting of pseudocereal grains increases the content and availability of nutrients [6,7,8], reduces the levels of antinutritional factors [9,10], and increases the amounts of bioactive compounds (soluble phenolic compounds [TSPCs]), γ-aminobutyric acid (GABA) and antioxidant activity (AA) [6,11,12]. Recently, there is a growing market for GABA-enriched foods due to their recognized health benefits such stress reduction and sleep enhancement [13] and their antihypertensive, anticancer, antidiabetic, anti-inflammatory, antioxidant, anti-allergy, and antimicrobial effects [14]. In this sense, sprouted grains with significantly higher content of GABA as compared to non-germinated grains have gained recognition as dietary sources of this bioactive non-protein amino acid [7,8]. The germination conditions are crucial for improving sprout quality and health-promoting properties. Therefore, a number of scientific papers published in the field have focused on the optimization of germination conditions to maximize the goals set in terms of nutritional quality [7,8,15,16]. According to Peñaranda et al. [4], most of the products launched with sprouted ingredients in the last years have been bakery products, snacks, and flours.

Biscuits are baked products that generally contain three major ingredients (flour, sugar, and fat), and have a low final water content (1–5 g/100 g) [17]. Biscuits comprise a major category of snacks by the virtue of their general acceptability, convenience, and shelf stability. In this sense, biscuits, which are broadly consumed, could be a good vehicle for bioactive compounds. The ability to fortify biscuits has led to them receiving more attention for formulating functional foods. For instance, recent studies have investigated the partial substitution of wheat flour in biscuits with orange peel powder [18] and bergamot by-products [19]. Innovations in biscuit recipes with sprouted pseudocereal flours to produce biscuits could be also a promising strategy that has not yet been fully addressed.

The pptimal formulation of new food products containing germinated grains is essential for better nutritional, sensorial, health, and technological attributes. For this purpose, the design of experiments (DoE), especially mixture design (MD) can help in the excellence to determine optimal formulations through predictive equations that allow the application of mathematical algorithms [20]. Among the most frequent applications of MDs in bakery products are formulations of gluten-free products, such as the formulation of bread using sorghum flour, rice flour, and millet flour [21,22].

The objective of this study was to develop biscuits with improved nutritional quality replacing refined wheat flour (WF) by binary blends of sprouted Andean grains: cañihua (*Chenopodium pallidicaule* Aellen), kiwicha (*Amaranthus caudatus* L.), and quinoa (*Chenopodium quinoa* Willd.). To this end, a simplex-centroid MD was used to study the effects of adding sprouted pseudocereal grains on PA, GABA, TSPC, and AA (as determined by ORAC assay) in the biscuit and identify the optimal formulation for increased content of bioactive compounds and AA and reduced levels of PA in biscuits. The effect of gastric and intestinal digestion on bioactive compounds, AA, PA, and starch hydrolysis was also evaluated.

## 2. Materials and Methods

### 2.1. Chemicals, Standards, and Reagents

Fast Blue BB (FBBB) [4-(benzoylamino)-2,5-dimethoxybenzenediazonium chloride hemi-(zinc chloride), bile extract porcine, pancreatin from porcine pancreas, pepsin from porcine gastric mucosa, α-amylase from human saliva, 2,2′-diazobis-(2-aminodinopropane)-dihydrochloride (AAPH), fluorescein, and GABA (>99% purity) were purchased from Sigma-Aldrich, Co. (St. Louis, MO, USA).

### 2.2. Grains, Sprouting, and Milling Processes

Pseudocereal grains cañihua (*Chenopodium pallidicaule* Aillen), kiwicha (*Amaranthus caudatus* L. var. Centenario), and quinoa (*Chenopodium quinoa* Willd. var. Pasankalla) originally from the Andean region were supplied by the Cereals and Native Grains Program of Universidad Nacional Agraria La Molina (Peru). Cañihua, kiwicha, and quinoa grains were harvested in 2019 from three different geographical areas. Cañihua was grown in the area of Suni (Puno, Perú) at 3827 m altitude. Kiwicha was grown in El Caserío de Huanchacpampa of Carhuaz (Ancash, Perú) at 2688 m altitude. Quinoa was grown in Junín (Jauja, Perú) at an altitude of 3335 m. Grains were stored at 20 °C in a dry container.

Grains were sprouted at optimal temperature and time to maximize the content of TSPC, GABA, and AA, as reported previously [7,8,12]. Sprouting parameters are summarized in Table S1. Briefly, 300 g of each seed type were disinfected for 30 min with 0.01% sodium hypochlorite before soaking in sterile water (1:5, *w*:*v*) at 23 °C for 7 h and then rinsed three to four times with tap water. Soaked seeds were covered by a moist filter paper and placed in a thermostatically controlled climatic chamber (model BJPX-HT400II, Maquilak, Jinan, China) with a water circulating system to maintain air humidity ≥ 90%.

Sprouted grains were dried in a climatic chamber at 40 °C for 30 h. These conditions were chosen based on our preliminary experiments. The drying temperature was set as low as 40 °C to avoid the thermal degradation of bioactive compounds while 30 h was the minimum time to reach 10% moisture in sprouts. Dried sprouts were milled in a MDNT-60XL grinding module and passed through a sieve with a 0.20 mm pore size (Torrh, Jarcon del Peru S.R.L., Junín, Peru). Three types of flour were obtained [sprouted cañihua flour (SCF), sprouted kiwicha flour (SKF), and sprouted quinoa flour (SQF)] and stored at 4 °C under vacuum in plastic bags.

### 2.3. Biscuit Making

Three types of biscuits were prepared using binary combinations of sprouted pseudocereal grains and wheat flour (WF):

- Biscuit 1: SQF, SKF, and WF. This biscuit was coded as BQK.
- Biscuit 2: SQF, SCF, and WF. This biscuit was coded as BQC.
- Biscuit 3: SKF, SCH, and WF. This biscuit was coded as BKC.

**Figure 1:**
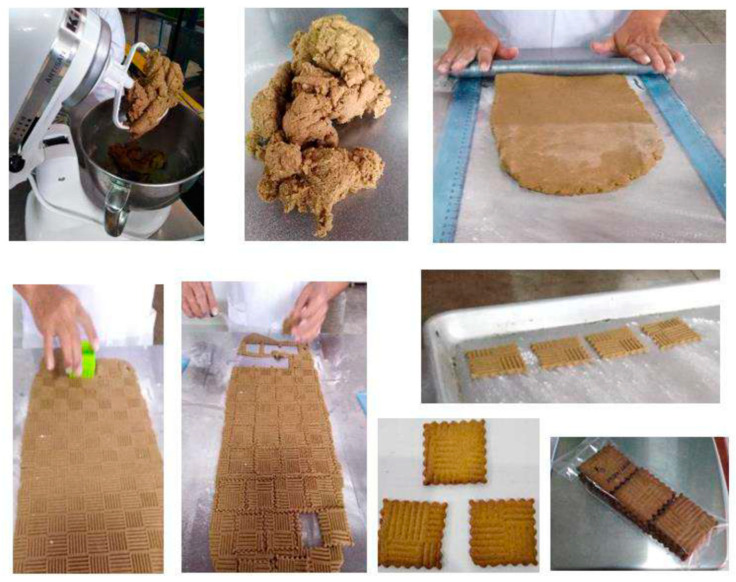
Biscuit elaboration process. From left to right: kneading; biscuit dough; dough sheeted with a rolling pin; biscuits were cut and placed in an oven tray; biscuits baked at 150 °C for 15 min; and wrapped in polypropylene plastic bags.

A control biscuit made up with 100% WF was prepared. WF (Nicolini, Alicorp S.A., Lima, Peru) was purchased on the market. For each biscuit, fourteen formulations were prepared according to a simplex centroid MD (Table S2). The biscuit making process is illustrated in Figure 1. For each formulation, to each 100 g of fresh dough, 53.5 g of flour, 15.5 g of blond sucrose (Yuyin, Lima, Perú), 11.6 g of water, 13.7 g of sunflower seed oil (Primor, Lima, Perú), 0.13 g vanilla cream (A&M, Lima, Perú), 0.89 g powdered skim milk (Anchor, Auckland, New Zealand), 0.13 g cinnamon powder (Sibarita, Lima, Perú), 0.18 g of baking powder (mixture of bicarbonate and weak acid) (Universal, Lima, Perú), and 0.31 g of salt (Lobos, Lima, Perú) were added. In the first mixing phase (cremation stage), blond sugar, oil, salt, vanilla cream, and water were added little by little and mixed by using a Kitchen-Aid Professional mixer (KPM5, KitchenAid, St. Joseph, MI, USA) with a dough hook (K45DH) for 10 min at speed 10. Finally, flour mix, milk powder, and cinnamon powder (0.25%) were added and mixed for another 2 min at speed 1. Subsequently, the dough was wrapped in a polypropylene plastic film and left to rest for 10 min; then, it was sheeted with a wooden rolling pin, and its height was adjusted to 3 mm with a regulating rolling pin. Biscuits were cut in a square with a biscuit cutter of 5 cm. Then, the biscuits were baked at 150 °C in an industrial oven (NOVA Max 1000, Lima, Peru) for 15 min. Three replicates for each formulation were made. After cooling, biscuits were sealed in polypropylene plastic bags before analysis and stored at 20 ± 2 °C for 2 weeks. To perform the in vitro digestion and subsequent analysis, samples were milled and stored under vacuum in polypropylene bags at −20 °C.

### 2.4. Simulated Gastrointestinal Digestion

INFOGEST 2.0 method [23] was performed to simulate the gastric and intestinal phase digestion of biscuits. Before the in vitro digestion, enzyme activities and bile concentration were determined. Briefly, 3 g of sample was mixed (1:1 ratio, *w*:*v*) with simulated salivary fluid containing 75 U/mL of salivary amylase and 0.3 M calcium chloride for 2 min at 37 °C in a Büchi B-491 heating bath (Marshall Scientific, Hampton, NH, USA). Subsequently, the pH value was quickly adjusted to 3 by adding 1 M HCl. The oral bolus was diluted (1:1 ratio, *v*:*v*) with simulated gastric fluid including 2000 U/mL pepsin solution and 0.3 M calcium chloride. In order to finish the gastric phase, pH was adjusted to 7 with 1 M sodium hydroxide and then simulated intestinal fluid containing 0.3 M calcium chloride, 800 U/mL pancreatin, and 10 mM bile were added to gastric chyme (1:1 ratio, *v*:*v*). Both gastric and intestinal phases were incubated for 2 h at 37 °C and 150 rpm in a G25 controlled environment incubator shaker (New Brunswick Scientific, Edison, NJ, USA). Finally, enzymes were inactivated by heating in a water bath (95 °C for 10 min). Digestion phases were freeze-dried (Virtis Company, Gardiner, NY, USA) and stored at −20 °C until further analysis. All phases were performed in duplicate.

### 2.5. Chemical Characterization of Raw and Sprouted Cañihua, Kiwicha, and Quinoa Flours

Moisture was determined by a gravimetric method (AACC 44-15A). Protein determination was carried out by the Dumas combustion method (AACC 46–13) and nitrogen conversion factor of 5.53 for pseudocereal flours according to ISO (International Organization for Standardization)/TS (Technical Specification) 16634-1 and ISO/TS 16634-2. Fat and ash were determined according to the standard AACC method 30–10 and 08–03, respectively [24]. The starch content in flours was measured using the K-TSTA-100A enzymatic total starch assay kit (Megazyme, Wicklow, Ireland). Proteins, fat, ash, and starch were expressed in g/100 g of dry weight (dw). The PA was measured using the K-PHYT enzymatic assay kit (Megazyme, Wicklow, Ireland) [25]. The total released phosphate was measured by a colorimetric technique at 655 nm using a microplate reader (BioTek Instruments, Winooski, VT, USA). Samples were analyzed in duplicate and the results were expressed in g/100 g of dw.

### 2.6. Extraction and Quantification of Total Soluble Phenolic Compounds (TSPCs)

TSPCs were analyzed by FBBB reaction according to Pico et al. [26] with slight modifications. A quantity of 50–100 mg of the milled sample was extracted with 1 mL of 80% methanol in 0.1% formic acid. The sample was vortexed and then incubated for 15 min at 30 °C and 2000 rpm (ThermoMixer Compact, Eppendorf AG, Hamburg, Germany). Subsequently, the sample was centrifuged for 5 min at 5 °C and 10,000 rpm (Centrifuge 5424 R, Eppendorf AG, Hamburg, Germany). The sample solution was collected and a second extraction cycle was performed with 1 mL of 70% acetone in 0.1% formic acid. The methanolic and acetone extracts were combined and the final volume was adjusted to 2 mL with deionized water. A volume of 1 mL of TPSC extract was mixed with 100 µL of freshly prepared FBBB reagent (0.1% in distilled water) and vortexed for 1 min. Immediately, the extract solution was shaken after adding 100 µL of 5% NaOH and incubated for 120 min in the dark at room temperature (20 °C). Finally, 200 µL of the incubated mixture was placed in a 96-well plate and absorbance was measured in triplicate at 420 nm using a Synergy HT microplate reader (BioTek Instruments, Winooski, VT, USA). Quantification of TSPCs was performed with linear calibration curves of gallic acid (0–225 µg/mL) that showed good linearity (R^2^ > 0.99). All analyses were performed in duplicate. Data were expressed as mg of gallic acid equivalents (GAE)/100 g of sample dw.

### 2.7. Determination of γ-Aminobutyric Acid (GABA)

Samples were extracted for 30 min by mechanical shaking of 200 mg of sample in 2 mL of 0.1 N HCl using a Thermomixer C (Eppendorf, Madrid, Spain) at 5 °C. Samples were centrifuged for 30 min at 5 °C and 8000× *g* (Centrifuge 5424 R, Eppendorf AG, Hamburg, Germany) and supernatants were filtered using a syringe filter with 0.22 μm nylon membranes. Analysis of GABA in the supernatant was performed by reverse-phase high-performance liquid chromatography (RP-HPLC) and UV detection after pre-column derivatization with 9-fluorenylmethoxycarbonyl chloride (FMOC) and *o*-phthaldialdehyde reagents (Agilent, Santa Clara, CA, USA). Chromatographic separations were carried out in an Agilent 1200 high-performance liquid chromatograph (Agilent, Santa Clara, CA, USA) equipped with a G1314B diode array detector (DAD) and a Zorbax Eclipse Plus C18 stationary phase column (4.6 × 150 mm, 3 μm). The mobile phase A was composed of 10 mM Na_2_HPO_4_:10 mM Na_2_B_4_O_7_, pH 8.2: 5 mM NaN_3_ and the mobile phase B consisted of acetonitrile:methanol:water (45:45:10, *v*:*v*:*v*). All mobile phase solvents were HPLC grade. Analysis were performed at 40 °C, with a flow rate of 1.5 mL/min and the following solvent gradient: 57% B in 20 min, 100% B in 20.1 min, 100% B in 23.5 min, 2% B in 23.6 min, 2% B in 25 min. The DAD detector was set to 338 nm (from 0–15 min) and 262 nm (from 15–30 min). External calibration was performed using standard solutions of GABA (Merck, Madrid, Spain) in the linear range between 10 and 1000 pmol/μL (R^2^ > 0.99). All analyses were performed in duplicate. Results were expressed as mg/100 g dw.

### 2.8. Determination of Oxygen Radical Antioxidant Capacity (ORAC)

The AA was determined by the oxygen radical absorbance capacity (ORAC) method described previously [15]. Briefly, the reaction was performed at 37 °C in 75 mM phosphate buffer at pH 7.4. The reaction mixture (200 μL) contained 180 μL of 70 nM fluorescein, 90 μL of 12 mM 2,2′-azobis(2-amidinopropane) dihydrochloride (AAPH) and 30 μL of diluted sample or standard (6-hydroxy-2,5,7,8-tetramethylchroman-2-carboxylic acid, Trolox, Hoffman-LaRoche, basel, Switzerland) at concentrations ranging from 1 to 160 μM. Reaction mixtures were placed in a black 96-well plate (Fisher Scientific, Hampton, VA, USA) and the fluorescence was read in a Synergy HT microplate reader (BioTek Instruments, Winowski, VT, USA) every minute at excitation and emission wavelengths of 485 and 520 nm, respectively. The equipment was controlled by Gen5™ software, version 1.1 (BioTek Instruments). Results were expressed as μmol Trolox equivalents (TE)/g dw.

### 2.9. Estimation of In Vitro Glycemic Index

To evaluate the in vitro rate of starch hydrolysis, the method described by Goñi et al. [27] was employed, with slight modifications based on Sanz-Penella et al. [28]. The hydrolysis index (HI) of the samples was calculated from the area under the curve (AUC) from 0 to 120 min as a percentage of the corresponding reference area (wheat bread; HI = AUC_sample_/AUC_wheat bread_ × 100). The estimated glycemic index (GI) was calculated using the equation GI = 0.549 × HI + 39.71. Analyses were carried out in triplicate.

### 2.10. Simplex Centroid Mixture Design

Flour formulation was optimized using the simplex centroid MD for biscuits BQK, BQC, and BKQ, respectively. The experimental formulations tested are shown in Table S2. In an experiment with q components, the proportions of the ingredients may be denoted by x_1_, x_2_, …, x_q_, where x_i_ ≥ 0 for i = 1, 2, …, q and ∑q_i_ = 1, x_i_ = 1, where x_i_ represents the proportion of the i-th component. This equation removes a degree of freedom from the proportions and the factor space is, therefore, a (q − 1)-dimensional regular simplex [20]. The design enabled us to approximate the experimental data (Y_obs_) with a response surface model represented in Equations (1)–(4):

Linear ŷ = ∑q_i_ = 1β_i_x_i_, (1)

Quadratic ŷ = ∑q_i_ = 1β_i_x_i_ + ∑_q−1_^i<j^∑q_j_β_ij_x_i_x_j_, (2)

Special cubic ŷ = ∑q_i_ = 1β_i_x_i_ + ∑_q__−__1_^i<j^∑q_j_β_ij_x_i_x_j_ + ∑_q−2_^i<j^∑_q__−__1_^j<k^∑q_k_β_ij_kx_i_x_j_x_k_, (3)

Full cubic: ŷ = ∑q_i_ = 1β_i_x_i_ + ∑_q−1_^i<j^∑q_j_β_ij_x_i_x_j_ + ∑_q−1_^i<j^∑q_j_δ_ij_x_i_x_j_(x_i_−x_j_) + ∑_q−2_ i < j∑_q−1_j < k∑q_k_β_ijk_x_i_x_j_x_k_, (4)

The parameter β_i_ represents the expected response to the pure blend x_i_ = 1 and x_j_ = 0 when j ≠ i. The term ∑q_i_ = 1β_i_x_i_ represents the linear blending portion. When curvature arises from non-linear blending between component pairs, the parameters βij, which represent either synergistic or antagonistic blending, will be different from zero [29].

The difference between the experimental data (Y_obs_) and model (Y_calc_) gives the residual (ε). For each response, the R^2^ (squared correlation coefficient) was calculated, which is the fraction of variation of the response explained by the model.

The response variables were PA, GABA, TSPC, and ORAC. Fourteen formulations were studied with different proportions of cañihua, kiwicha, and/or quinoa, as shown in Table S2.

### 2.11. Statistical Analysis

Three replicates of each experimental formulation were performed while each parameter was analyzed twice per replicate (a data set of 6 values was obtained per sample). Results were expressed as mean ± standard deviation. The *t*-test (two data sets for comparison of groups treatment vs. control) with post hoc Dunnet’s test or one-way analysis of variance (ANOVA, three or more data sets) with Bonferroni post hoc test was conducted assuming Gaussian normal distribution and homogeneity of variances. Regression models for the PA, GABA, TSPC, and ORAC were generated using DoE in Statistica v.9.0 software (Stasoft, Tulsa, OK, USA). The ANOVA of regression models was performed to choose the most significant model (*p* ≤ 0.05) and the best fit (R^2^). Response surfaces and desirability methodology were used to identify optimal formulation for each biscuit type.

## 3. Results and Discussion

### 3.1. Effect of Germination on Nutritional Composition of Cañihua, Kiwicha, and Quinoa Flours

The chemical composition of cañihua, kiwicha, and quinoa flours obtained from raw (CF, KF, and QF, respectively) and sprouted (SCF, SKF, and SQF, respectively) grains is given in Table 1. Refined WF showed the highest starch content (75 g/100 g dw) compared to wholegrain (40–48 g/100 g dw) and sprouted (17–41 g/100 g dw) pseudocereal flours. This may be particularly interesting as the incorporation of these pseudocereal flours in baked products could reduce their GI values. Sprouted pseudocereal flours showed a reduced content of total starch compared to their ungerminated grains. Sprouting initiates the de novo synthesis of starch-degrading enzymes, such as α-amylase and α-glucosidase, which are responsible for the enzymatic hydrolysis of starch [30] and increased content of mono-, di- and oligosaccharides, as reported for cañihua [12], kiwicha [31], and quinoa sprouts [32]. In line with our results, differences in the magnitude of starch reduction after sprouting of pseudocereal grains were observed. For instance, the total starch content decreased by 14% to 17% in red and white quinoa sprouted for 48 h (temperature not specified) [33], whereas a higher reduction (approximately 72%) was observed for a commercial quinoa grain (Quinoa Marche Srls, Jesi, Ancona, Italy) germinated for 24 h at 16.5 °C [32]. This variation could be attributed to differences in the genotype and germination conditions used [30].

The protein content of wholegrain and sprouted pseudocereals flours was higher than wheat (12 g/100 g dw) and ranged from 14 g/100 g dw to 21 g/100 g dw (Table 1) according to literature data [1,2]. Comparative analysis revealed that WF had the lowest protein content compared with wholegrain and germinated pseudocereal flours. In general, the sprouting of cañihua and kiwicha did not cause relevant changes in total protein content (7% and 4% reduction), whereas a 20% reduction in protein content was observed for SQF with respect to QF. In general, while sprouting lead to protein hydrolysis by the activation of protease and endopepdidases, the nitrogen balance is commonly maintained during seed germination and sprout development [30]. For example, Perri et al. [32] reported that protein content was not significantly affected by the sprouting process in wheat, barley, chickpea, lentil, and quinoa germinated from 24 h to 120 h at 16.5 °C. However, some studies reported a significant decrease, from 5.2% to 12.5%, in the protein content in the quinoa varieties of Pasankalla roja and INIA Salcedo after germination for 48 h at 20–25 °C [11]. In contrast, other studies have reported an increase in protein content from 8% to 22.5% in sprouted quinoa Chulpi and kiwicha Oscar Blanco [6], and quinoa Negra Collana [11]. These different effects of germination on protein content have been ascribed to seed metabolism during germination. The decrease in protein content was attributed to protein hydrolysis of soluble peptides and amino acids that may leach in the soaking water, while the increase can probably be explained by the biosynthesis of new proteins or the loss of carbohydrates through respiration [34].

The three pseudocereal grains and sprouts contain a comparable fat content (5.1–6.5 g/100 g dw), but it was significantly higher than refined WF (0.6 g/100 g dw) (Table 1), in agreement with the literature [1,2]. The fat content of SCF and SKF was similar to ungerminated counterparts in line with results reported in the literature for wheat, barley, chickpea, lentil, and quinoa germinated for 24–120 h at 16.5 °C [32,33]. In contrast, SQF had a 1.4-fold higher fat content compared to QF. The same results were found by Darwish et al. [9] when the fat content in quinoa seeds was compared with sprouted quinoa for 72 h at 25 °C. In this study, the lipid increase caused by the sprouting process in quinoa was ascribed to changes in the macronutrient distribution that led to a significant reduction in total starch and protein contents, similar to our results. The quality of the lipids present in sprouted quinoa and kiwicha flours was characterized by other authors, confirming their high content of unsaturated fatty acids with linoleic acid being the most predominant followed by oleic, palmitic, and linoleic acids [33]. The low value for the linoleic acid/alpha-linolenic acid ratio of sprouted pseudocereal flours may positively impact health; therefore, the consumption of food formulation incorporating these flours may promote healthy eating and prevent cardiovascular diseases [35].

As compared to refined WF, pseudocereal flours showed significantly higher ash content (Table 1). Wholegrain pseudocereal flours displayed a similar ash content (2.5–2.8% dw), which was not influenced by the sprouting process (2.3–2.9 g/100 g dw). In line with our results, Perri et al. [32] observed nonsignificant differences when flours obtained from wheat, barley, chickpea, lentil, and quinoa grains were compared before and after the sprouting process. Other studies have reported significant decreases, ranging from 5% to 37% reduction, in the ash content after malting of different varieties of quinoa [6,11]. Bhinder et al. [10] observed the loss in Zn, K, and Mg after malting of white and black quinoa while Fe increased for all quinoa varieties. Mineral reduction in malted quinoa was attributed to mineral leaching during steeping or loss during deculming (root removal). Contrasting results were recently reported by other authors who described an increase in ash content after germination in *A. caudatus* (kiwicha), *A. quitensis* [31], and quinoa [36], which was similar to that explained by the reduction of total solids and the subsequent proportional increase in mineral content.

One of the most abundant minerals in amaranth and quinoa species is phosphorous (P) [2]. P amounts ranged between 4433 and 5889 mg/kg in amaranth and 4287–5738 mg/kg in quinoa stored as PA in seeds [1]. Due to its mineral chelating activity, PA is considered an antinutritional factor and reduces the bioavailability of divalent ions (Ca^2+^, Fe^2+^, Mg^2+^, Mn^2+^, and Zn^2+^) during digestion. A variation in the PA content of the studied flours was observed (Table 1). KF stood out for its higher PA content (1.2 g/100 g dw), followed closely by CF (1.1 g/100 g dw) and QF (0.9 g/100 g dw) in line with reported PA values in quinoa (0.2–1.0 g/100 g dw) and amaranth (0.3–0.6 g/100 g dw) [3,9,10,36,37]. Flours from sprouted pseudocereal grains showed a reduced (for SCF) or similar PA content (for SKF and SQF) with respect to wholegrain flours and WF. Several studies have similarly reported lower PA amounts (decreased by 16.4–50%) in flours obtained from sprouted and malted quinoa [9,10,33]. During germination, seed phytases are activated, de novo synthesized, and secreted to make phosphate, mineral elements, and myoinositol available for plant growth and development [30]. Thus, controlled grain sprouting increases the bioaccessibility of mineral elements. Comparing with sprouted pseudocereal flours, SCF and SQF had a 1.4-fold lower PA content than SKF.

GABA is a bioactive non-protein amino acid with multiple health effects, such as inhibition of tumor cell proliferation, reduction of blood pressure, improvement in brain function, stimulation of immune system, and prevention of diabetes [38]. While the GABA content in WF, CF, KF, and QF was low (13, 24, 37, and 33 mg/100 g dw, respectively), flours from sprouted pseudocereal grains showed a noticeably higher GABA content (100–218 mg/100 g dw) (Table 1). The highest increase in GABA after sprouting was observed in cañihua (8.9-fold increase), followed by quinoa (6.1-fold increase) and kiwicha (2.7-fold increase). GABA is produced during seed germination by the decarboxylation of L-glutamic acid by the action of glutamate decarboxylase (GAD) [39]. Germination causes partial hydrolysis of proteins, increasing the availability of free glutamic acid, which, together with the activation of GAD [39], are responsible for GABA enhancement in sprouts obtained at higher temperatures and longer germination times. Compared with cereals, the GABA content of sprouted pseudocereal flours reported in this study was higher than WF (7–25 mg/100 g) and the reported values for sprouted oat (54.92 mg/100 g), sprouted barley (81–186 mg/100 g), and sprouted brown rice (35 to 80 mg/100 g of dm) [15,16,30]. Other strategies consisting of steeping the kernels at 30 °C in lactic or citric acid solutions (pH 3.0 to 5.6) prior to germination for 24 to 48 h at 30 to 35 °C increased GABA content in sprouted brown rice up to 120 to 130 mg/100 g dm [30], although these values were still lower to those found in SCF and SQF. It is noteworthy that GABA is very heat-stable and it is not degraded during kilning/drying [40].

TSPC content differed among flours from different pseudocereal grains (Table 1). The highest TSPC content was observed for QF (525.5 mg GAE/100 g), followed by CF (314.4 mg GAE/100 g), KF (149.3 mg/100 g), and refined WF (44.3 mg GAE/100 g). These values were in the range reported in the literature for quinoa (14.37–597 mg/100 g) [3], cañihua (170–740 mg GAE/100 g) [12,41], and amaranth (123–155 mg GAE/100 g) [42]. The germination process caused a significant enhancement of TSPC in sprouted flours. The comparative analysis of flours revealed that SQF (612.8 mg GAE/100 g) stood out for its greater TSPC content. A trend for increased TSPCs was also reported in earlier studies after sprouting of cañihua [12], amaranth species (including kiwicha) [42], and quinoa [43]. Germination activates glucanases and cinnamoyl and feruloyl esterases that degrade the cell wall and contribute to the release of bound phenolic forms, thereby increasing TSPCs in grains after sprouting [30]. Moreover, de novo synthesis of phenolic compounds during sprouting by activation of the shikimate and phenylpropanoid pathways could also be responsible for higher TSPC content after grain sprouting, as has been demonstrated in wheat [44].

Higher ORAC was found for CF and QF flours as compared to KF (Table 1). The ORAC values for this study fell within the range of reported values for pseudocereal flours found in the literature (1.8–70.9 μmol TE/g for cañihua, 32.8–51.4 μmol TE/g for amaranth and 8–60 for quinoa) [12,42,43]. In general, sprouting increases the AA of pseudocereals. Various researchers have shown between 1.2 and 6.4 fold increases in the AA after germination of cañihua [12], kiwicha [31] and quinoa [43] when sprouted for 48 to 120 h at 15 to 28 °C. The higher AA in sprouted grains is mainly attributable to the accumulation of vitamin E and polyphenols [30].

### 3.2. Effect of the WF Replacement by Sprouted Pseudocereal Flours on PA, GABA, TSPCs, and AA in Biscuits

When optimizing a baking product formulation, the objective is to minimize PA content as it is the main factor that affects negatively mineral bioavailability. Table 2 shows the PA content of control biscuits (100% refined WF) and those formulated with a mixture of refined WF (60–80%) and two types of sprouted pseudocereal flours (20–40%). The PA content of all studied formulations varied within a similar range (0.15–0.33 g/100 g, 0.17–0.25 g/100 g, 0.14–0.25 g/100 g for BQK, BQC, and BKC, respectively). As compared to control biscuit (0.04%), all studied formulations had higher amounts of PA. This means that inclusion of sprouted pseudocereal flours increased PA; specifically, the highest PA content was observed in biscuits composed of 40% sprouted pseudocereal flours, especially in BQK (0.3%), which was prepared from flours (SQF and SKF) with higher PA levels (Table 1). Compared to other cereal products, biscuits developed in this study had lower PA levels than white and wholegrain wheat bread (0.43–1.05%), oatmeal (0.8–1.03%), or within the range of whole meal rye bread (0.03–0.41%) and rice (0.06–2.20%) [45].

The GABA content of the different flour formulations varied from 2.4–4.5 mg/100 g, 2.6–6.5 mg/100 g, and 2.5–4.1 mg/100 g for BQK, BQC, and BKC, respectively (Table 2). These values were in the range reported for cereal-based enriched foods (2.2–16.2 mg/100 g) [46] and malted sorghum cookies (2.4–5.9 mg/100 g) [47]. The incorporation of sprouted pseudocereal flours in the biscuit formulation improved the GABA content by as much as 9-fold. The comparative analysis of the three types of biscuits (BQK, BQC, and BKC) revealed that the formulations containing higher proportions of SQF stood out with regard to GABA content, as this type of flour was the richest in this non-protein amino acid (Table 1). There is no official recommendation for GABA intake, although clinical evidence from 16 studies that examined the effect of GABA in different matrices on mild hypertension at doses ranging from 10 to 12 mg/day for up to 12 weeks or 120 mg of GABA/day for 12 weeks reported that the ingestion of GABA was associated with a moderate drop (≤10% change) in blood pressure, which returned to baseline level a few days after the participants stopped taking the product containing GABA [48]. Therefore, a serving of 50 g of biscuits could provide a modest contribution to reaching the effective daily dose associated to health effects.

A significant variation was also observed for the TSPC content of biscuits prepared with all experimental formulations. In particular, TSPC content oscillated between 128.9–246.5 mg GAE/100 g, 204.4–486.9 mg GAE/100 g, and 127–328.6 mg GAE/100 g for BQK, BQC, and BKC, respectively (Table 2). The substitution of WF with sprouted pseudocereals flour caused a 9-fold increase in the TSPC content as compared to control biscuits (52.6 mg GAE/100 g). Higher increases in TSPC content were observed in BQC at the highest level of substitution (40% total SQF and SCF mixture). Regardless of formulation, the phenolic content of biscuits from this study was higher than that reported in the literature for biscuits made with 100% wholegrain quinoa (142 mg GAE/100 g) [49,50], but lower than for biscuits made from sprouted *Chenopodium album* flour (671 mg GAE/100 g) [51]. Regular dietary intake of polyphenols, approximately 1–2 g per day, has been associated with chronic disease prevention [52]. Although there is no official recommendation for phenolic compounds, it is worth noting that a serving of 50 g of BQC (40% substitution) could provide almost 25% of the reported mean daily intake (1 and 1.2 g/d) [53].

A large variation was found for the AA in biscuits, and it was significantly influenced by flour formulation (Table 2). This parameter ranged from 51.3–91.8 μmol TE/g, 56.1–138.4 μmol TE/g, and 59.8–114.5 μmol TE/g for BQK, BQC, and BKC, respectively (Table 3). These values are higher than the AA of control biscuits (25.5 μmol TE/g); therefore, the incorporation of sprouted pseudocereal flours in the formulation of biscuits significantly improved their antioxidant potential, as reported in other studies [51,54]. Among the three types of biscuits, BQC (40% substitution) was characterized to have the highest AA, which could be attributed to the higher AA of SCF and SQF compared to SKF, in accordance with its higher phenolic content (Table 1).

### 3.3. Modelization of Flour Formulation Effects on PA, GABA, TSPCs, and AA in Biscuits

**Figure 2:**
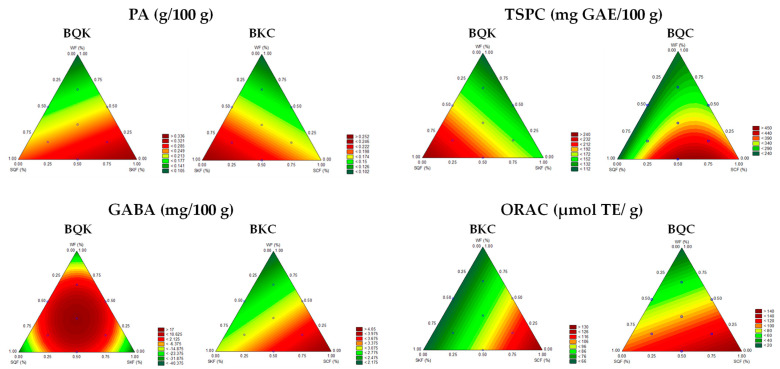
Contour plots of predicted PA, GABA, TSPCs, and ORAC where X_1_, X_2_, and X_3_ are normalized to 100%. Abbreviations: BQK, biscuits formulated with sprouted quinoa (X_1_), sprouted kiwicha (X_2_), and wheat flour (X_3_); BQC, biscuits formulated with sprouted quinoa (X_1_), sprouted cañihua (X_2_), and wheat flour (X_3_); BKC biscuits formulated with sprouted kiwicha (X_1_), sprouted cañihua (X_2_), and wheat flour (X_3_); GABA, γ-aminobutyric acid; GAE, gallic acid equivalents; ORAC, oxygen radical absorbance capacity; PA, phytic acid; TSPCs, total soluble phenolic compounds; TE: Trolox equivalents.

The canonical equations adjusted to experimental data for the dependent variables PA, GABA, TSPCs, and AA for BQK, BQC, and BKC are presented in Table 3; only terms with coefficients considered significant at the 5% level are provided. ANOVA was performed to confirm the confidence of prediction of the regression models. Significant models at the 5% level with a coefficient of determination (R^2^) higher than 0.7 were accepted for predictive purposes. Figure 2 shows the contour regions corresponding to the predictive equations for PA, GABA, TSPCs, and AA.

The predictive equations for PA content indicated that this parameter was positively influenced by sprouted pseudocereal flours (Table 3), particularly by the addition of SKF alone for both BQK and BKC (Figure 2). GABA content had significant positive linear and interaction coefficients (particular in the case of BQK) (Table 3), indicating that addition of sprouted pseudocereal flours (especially the mixture of SQF/SKF for BQK or SCF alone for BKC) increased the amounts of this bioactive compound (Figure 2). For TSPCs, all linear and interaction terms (in particular the case of BQC, Table 3) were significantly positive, suggesting that this parameter increased when the replacement rate was increased for sprouted pseudocereals flours (particularly SQF alone for BQK and SCF alone and blends with SQF for BQC, Figure 2). For the ORAC, all linear terms were significantly positive (Table 3), indicating that the addition of sprouted pseudocereal flours increased the AA in biscuits, especially when SCF was included in the formulation for BKC and BQC (Figure 2). The fact that SCF and SQF stood out in both biscuits may be because of their higher content of phenolic compounds (Table 1).

From a practical point of view, multiresponse optimizations were obtained by the application of a desirability function (D) in which the following criteria were selected: (1) to maximize the GABA, TSPCs, and AA of biscuits and to (2) minimize PA content. Table 4 shows the optimal formulations for the three types of biscuits. For BQK, the optimal formulation consists of 21% SQF, 16% SKF, and 63% WF, for which the predicted PA, GABA, and TSPCs were 0.24 g/100 g, 13.6 mg/100 g, and 177.9 mg GAE/100 g, respectively. For BQC, the optimal formulation was 15% SQF, 25% SCF, and 60% WF, to reach a maximum TSPC content of 472.4 mg GAE/100 g and an AA as high as 135.9 μmol TE/g. For BKC, the optimal formulation was 5% SKF, 23% SCF, and 72% WF to keep PA as low as 0.15 g/100 g, while simultaneously GABA and ORAC were predicted to reach up to 3.4 mg/100 g and 106.5 μmol TE/g.

To validate the model, the optimal formulation of BQC (selected for its highest D value), consisting of 15% SQF, 25% SCF, and 60% WF, was prepared in triplicate. Its nutritional values, in terms of GABA, TSPCs, AA, and PA, are presented in Table 5, and were compared with the predicted values in Table 4. The experimental values for TSPCs and ORAC varied by less than 5% (Table 4); therefore, the model was validated. A comparative analysis of bioactive compounds, AA, and PA levels was also carried out between the optimized formulation for BQC and the control biscuit (100% refined WF) (Table 5). The incorporation of SQF and SCF significantly increased all parameters studied.

### 3.4. In Vitro Digestion Analysis

Table 5 shows the fate of the control biscuit and BQC (optimal formulation) by observing the PA, GABA, TSPCs, and AA during gastric and intestinal digestion using the INFOGEST in vitro digestion model. For both biscuits, PA content remained unchanged after gastric digestion, but was significantly increased at the end of intestinal phase, reaching a higher concentration in control biscuit than in BQC. It is known that, in vivo, PA degradation might occur in the stomach due to activation of endogenous phytases in the food matrix in the acidic environment of the stomach or in the colon as consequence of the lower pH due to the fermentation of dietary fibers by specific microbiota species with a high phytase activity [55].

Similar to PA, changes in GABA concentration took place only at the end of intestinal phase, with a significant increase observed for the control and BQC, respectively (Table 5). Nonetheless, the amounts of bioaccessible GABA at the end of intestinal digestion were greater for BQC as compared to the control. The resistance of GABA to the gastric environment (pH 1.2) and intestinal conditions was also reported during the simulated gastrointestinal digestion of a GABA-rich yoghurt [56]. In another study, Dala-Paula et al. [57] indicated that GABA content increased after the in vitro digestion of chocolate due to digestive enzymes, which agreed with our results.

Nonsignificant differences were observed for TSPCs at the end of gastric digestion of BQC, while a small increase was seen for the control biscuit (Table 5). As digestion progressed, the bioaccessible amount of TSPCs markedly increased at the end of intestinal phase for both samples and BQC was notable for its higher TSPC content as compared to the control biscuit. Consistently with TSPC, AA increased at the end of gastric and intestinal digestion in both samples, with BQC having higher values than the control biscuit. Therefore, these results indicated that the enrichment of biscuits in antioxidant compounds (for instance TSPCs) was translated into increased AA. Similar results were reported by Hidalgo et al. [58], who observed increased levels of TSPCs and increased AA in breads after intestinal digestion, although they did not find differences in the total phenolic content and the AA in the intestinal in vitro digests among different bread formulations (wheat, eikorn, and eikorn mixture with quinoa, buckwheat, and amaranth).

Moreover, the starch digestion kinetic and GI of optimized formulation for BQC was compared to control biscuit and white wheat bread (Table 5, Figure S1). The in vitro starch hydrolysis was evaluated by plotting the percentage of starch hydrolyzed as a function of digestion time (Figure S1). The hydrolysis index (HI) and area under the curve (AUC) was calculated for each sample from the corresponding starch hydrolysis curves (Table 5). The highest HI and AUC after white bread (100 ± 3.16 and 31,642.5 ± 999.3, respectively; data not shown) were found for the control biscuits followed by BQC, which had significantly lower values. Values for predicted GI were calculated in relation to white bread. The addition of SQF and SCF led to a significant decrease of GI in BQC (81.4) compared to the control biscuit (87.7) and white bread (94.6) (Table 5). As compared with literature data, BQC has a lower GI than biscuits made of rice flour, wheat:maize (2:1), rice flour and waxy rice starch, and commercial digestive biscuits, although it was higher than biscuits made of wholegrain and malted tartary buckwheat, bean and maize, foxtail millet and wheat (45:55), and banyard millet and wheat (45:55) [17]. Similar results have been reported recently by Wang et al., who observed that 40% addition of quinoa flour led to a 17% reduction in maximum starch digestion compared to wheat breads [59]. The reduced GI in BQC could be ascribed to the addition of SQF and SCF with increased protein and fiber content, which may have contributed to the reduction in the glycemic load and the accessibility of α-amylase to starch [17]. Moreover, the enrichment of biscuits in phenolic compounds could result in the inhibition of α-amylase activity [60].

## 4. Conclusions

This work determined that is possible to produce biscuits with improved nutritional and health benefits through the optimization of flour formulations based on the reduction of refined WF in favor of the incorporation of sprouted pseudocereals grains of Andean origin (kiwicha, quinoa, and cañihua). This strategy, joined to the use of statistical tools such as mixture design and multiresponse optimization, allowed biscuits to be obtained with higher content of GABA, TSPCs, AA, and lower GI and PA. Moreover, this study demonstrated that these nutritional benefits provided by the replacement of WF by sprouted pseudocereal flours persisted upon digestion. Considering the popularity of biscuits around the world, this information should be taken into account to transform biscuits into a more nutritive and healthier product to contribute to the improvement of public health.

## Figures and Tables

**Table 1:** Chemical composition (expressed as dry weight, dw) of cañihua, kiwicha, and quinoa flours from wholegrains and sprouted grains.

Parameters	WF	CF	SCF	KF	SKF	QF	SQF
Starch (g/100 g dw)	75.07 ± 0.97 ^f^	46.66 ± 0.45 ^d^	41.21 ± 1.47 ^c^	48.33 ± 0.93 ^d^	32.45 ± 0.85 ^b^	40.98 ± 0.88 ^c^	16.97± 0.85 ^a^
Protein (g/100 g dw)	12.26 ± 0.06 ^a^	20.61 ± 0.26 ^g^	19.11 ± 0.27 ^f^	16.05 ± 0.14 ^d^	15.38 ± 0.11 ^c^	16.87 ± 0.13 ^e^	13.52 ± 0.26 ^b^
Fat (g/100 g dw)	0.61 ± 0.04 ^a^	6.18 ± 0.04 ^d^	6.23 ± 0.25 ^d^	5.10 ± 0.08 ^b^	5.86 ± 0.38 ^cd^	5.21 ± 0.07 ^bc^	6.55 ± 0.11 ^e^
Ash (g/100 g dw)	0.55 ± 0.05 ^a^	2.76 ± 0.13 ^c^	2.68 ± 0.05 ^c^	2.66 ± 0.19 ^bc^	2.85 ± 0.08 ^c^	2.48 ± 0.17 ^bc^	2.29 ± 0.08 ^b^
PA (g/100 g dw)	0.09 ± 0.01 ^a^	1.17 ± 0.02 ^b^	0.88 ± 0.01 ^b^	1.23 ± 0.02 ^c^	1.24 ± 0.02 ^c^	0.90 ± 0.02 ^b^	0.93 ± 0.02 ^b^
GABA (mg/100 g dw)	12.96 ± 0.35 ^a^	24.34 ± 4.83 ^b^	217.98 ± 1.48 ^d^	37.38 ± 1.58 ^b^	100.00 ± 22.45 ^c^	32.98 ± 4.42 ^b^	202.54 ± 32.05 ^d^
TSPC (mg GAE/100 g)	44.27 ± 2.43 ^a^	314.39 ± 22.38 ^d^	386.12 ± 27.83 ^e^	149.27 ± 1.80 ^b^	244.72 ± 2.09 ^c^	525.5 ± 38.14 ^f^	612.81 ± 13.25 ^g^
ORAC (μmol TE/g)	20.64 ± 2.71 ^b^	48.74 ± 5.98 ^b^	114.92 ± 14.17 ^c^	10.23 ± 2.16 ^a^	35.44 ± 4.55 ^d^	46.62 ± 3.53 ^b^	45.30 ± 3.96 ^d^

Data are mean ± standard deviation of three replicates. Different letters denote statistical differences among samples (ANOVA, Bonferroni *post hoc* test, *p* ≤ 0.05). Abbreviations: CF, cañihua flour; dw, dry weight; GABA, γ-aminobutyric acid; GAE, gallic acid equivalents; KF, kiwicha flour; ORAC, oxygen radical absorbance capacity; PA, phytic acid; QF, quinoa flour; SCF: sprouted cañihua flour; SKF, sprouted kiwicha flour; SQF, sprouted quinoa flour; TSPC, total soluble phenolic compounds; TE: Trolox equivalents; WF: refined wheat flour.

**Table 2:** Effect of flour formulation on PA, GABA, TSPC, and AA (as determined by ORAC assay) in biscuits.

Biscuit Type	Recipe No.	Proportion of Flours ^a^			PA (g/100 g)	GABA (mg/100 g)	TSPC (mg GAE/100 g)	ORAC (μmol TE/g)
		Sprouted Pseudocereal Flour 1 (X_1_)	Sprouted Pseudocereal Flour 2 (X_2_)	Wheat Flour (X_3_)				
BQK	1	15	15	70	0.22 ± 0.00 ^c^	4.01 ± 0.06 ^de^	148.38 ± 3.80 ^bcd^	57.03 ± 1.39 ^bc^
	2	20	20	60	0.27 ± 0.01 ^de^	4.41 ± 0.11 ^e^	192.96 ± 1.69 ^fgh^	72.43 ± 6.25 ^def^
	3	5	20	75	0.23 ± 0.01 ^c^	2.44 ± 0.16 ^b^	133.00 ± 0.68 ^bc^	52.50 ± 6.27 ^b^
	4	20	20	60	0.32 ± 0.01 ^fg^	3.62 ± 0.26 ^cd^	222.30 ± 4.16 ^hi^	91.76 ± 1.94 ^g^
	5	5	20	75	0.22 ± 0.01 ^c^	2.54 ± 0.05 ^b^	125.71 ± 5.56 ^b^	59.83 ± 2.33 ^bc^
	6	5	20	75	0.22 ± 0.00 ^c^	2.50 ± 0.04 ^b^	128.86 ± 3.74 ^b^	52.39 ± 0.50 ^b^
	7	20	20	60	0.33 ± 0.01 ^g^	3.37 ± 0.04 ^c^	246.49 ± 15.01 ^i^	75.81 ± 2.44 ^f^
	8	10	25	65	0.29 ± 0.00 ^ef^	3.73 ± 0.00 ^cd^	160.46 ± 4.87 ^cde^	52.71 ± 1.50 ^b^
	9	20	5	75	0.16 ± 0.01 ^b^	3.85 ± 0.18 ^cd^	165.11 ± 4.65 ^def^	74.57 ± 5.01 ^ef^
	10	20	5	75	0.17 ± 0.00 ^b^	3.41 ± 0.07 ^c^	188.39 ± 16.35 ^efg^	63.03 ± 5.02 ^bcde^
	11	10	10	80	0.18 ± 0.02 ^b^	2.63 ± 0.02 ^b^	151.36 ± 9.06 ^bcd^	53.98 ± 2.48 ^bc^
	12	25	10	65	0.24 ± 0.01 ^cd^	4.49 ± 0.08 ^e^	212.15 ± 2.73 ^gh^	66.14 ± 5.52 ^cdef^
	13	20	5	75	0.22 ± 0.01 ^c^	4.12 ± 0.18 ^de^	222.31 ± 1.08 ^hi^	60.01 ± 4.08 ^bcd^
	14	25	10	65	0.15 ± 0.01 ^b^	3.38 ± 0.01 ^c^	175.10 ± 3.36 ^def^	51.30 ± 4.32 ^b^
BQC	1	15	15	70	0.19 ± 0.00 ^de^	3.50 ± 0.01 ^bcd^	409.48 ± 38.06 ^ef^	95.46 ± 2.45 ^c^
	2	20	20	60	0.25 ± 0.01 ^f^	4.66 ± 0.06 ^de^	486.86 ± 15.14 ^f^	133.28 ± 2.65 ^d^
	3	5	20	75	0.18 ± 0.00 ^cde^	4.01 ± 0.02 ^cde^	334.40 ± 2.85 ^de^	91.60 ± 6.44 ^c^
	4	20	20	60	0.23 ± 0.00 ^f^	4.58 ± 0.28 ^de^	441.61 ± 14.63 ^f^	138.39 ± 2.03 ^d^
	5	5	20	75	0.15 ± 0.00 ^b^	4.06 ± 0.08 ^cde^	320.15 ± 26.96 ^cd^	84.49 ± 0.25 ^c^
	6	5	20	75	0.17 ± 0.00 ^bcd^	3.63 ± 0.03 ^bcde^	280.14 ± 2.58 ^bcd^	89.03 ± 4.07 ^c^
	7	20	20	60	0.25 ± 0.00 ^f^	4.94 ± 0.22 ^e^	428.78 ± 6.39 ^f^	136.25 ± 2.19 ^d^
	8	10	25	65	0.23 ± 0.00 ^f^	3.60 ± 0.41 ^bcde^	411.83 ± 21.66 ^ef^	98.35 ± 8.20 ^c^
	9	20	5	75	0.18 ± 0.00 ^cde^	3.18 ± 0.02 ^bc^	204.43 ± 5.60 ^b^	67.38 ± 2.92 ^b^
	10	20	5	75	0.19 ± 0.00 ^cde^	3.12 ± 0.03 ^bc^	236.8 ± 0.310 ^b^	61.64 ± 5.84 ^b^
	11	10	10	80	0.17 ± 0.00 ^bc^	2.61 ± 0.02 ^b^	275.67 ± 3.05 ^bcd^	56.06 ± 5.06 ^b^
	12	25	10	65	0.24 ± 0.00 ^f^	4.58 ± 0.00 ^de^	339.32 ± 24.67 ^de^	90.22 ± 9.48 ^c^
	13	20	5	75	0.20 ± 0.01 ^e^	3.98 ± 1.03 ^cde^	247.89 ± 19.14 ^bc^	56.68 ± 5.22 ^b^
	14	25	10	65	0.23 ± 0.00 ^f^	6.47 ± 0.02 ^f^	325.52 ± 24.66 ^cd^	92.66 ± 2.98 ^c^
BKC	1	15	15	70	0.15 ± 0.01 ^bc^	3.32 ± 0.05 ^ef^	228.92 ± 16.62 ^def^	93.69 ± 1.03 ^ef^
	2	20	20	60	0.22 ± 0.01 ^e^	3.36 ± 0.08 ^ef^	328.63 ± 0.00 ^g^	98.96 ± 14.25 ^fg^
	3	5	20	75	0.15 ± 0.00 ^bc^	3.32 ± 0.09 ^ef^	260.76 ± 7.13 ^ef^	114.45 ± 2.37 ^g^
	4	20	20	60	0.21 ± 0.01 ^e^	3.64 ± 0.00 ^f^	279.73 ± 25.94 ^fg^	106.35 ± 3.91 ^fg^
	5	5	20	75	0.14 ± 0.00 ^b^	3.06 ± 0.03 ^cde^	230.75 ± 19.53 ^def^	106.14 ± 1.32 ^fg^
	6	5	20	75	0.14 ± 0.00 ^b^	3.23 ± 0.07 ^e^	287.82 ± 20.39 ^fg^	89.74 ± 2.97 ^def^
	7	20	20	60	0.25 ± 0.00 ^f^	4.11 ± 0.06 ^g^	236.09 ± 22.07 ^def^	99.16 ± 1.75 ^fg^
	8	10	25	65	0.17 ± 0.00 ^d^	3.32 ± 0.04 ^ef^	185.48 ± 13.65 ^bcd^	100.34 ± 3.40 ^fg^
	9	20	5	75	0.22 ± 0.01 ^e^	2.58 ± 0.05 ^b^	285.27 ± 8.29 ^fg^	68.19 ± 2.21 ^bc^
	10	20	5	75	0.16 ± 0.00 ^cd^	2.80 ± 0.06 ^bcd^	127.11 ± 4.57 ^b^	80.44 ± 4.35 ^cde^
	11	10	10	80	0.14 ± 0.00 ^b^	2.52 ± 0.10 ^b^	167.04 ± 13.22 ^bc^	62.95 ± 0.94 ^b^
	12	25	10	65	0.14 ± 0.00 ^b^	2.71 ± 0.22 ^bc^	240.33 ± 3.93 ^def^	67.51 ± 0.33 ^bc^
	13	20	5	75	0.18 ± 0.01 ^d^	2.46 ± 0.06 ^b^	147.29 ± 5.00 ^b^	59.81 ± 1.33 ^b^
	14	25	10	65	0.26 ± 0.01 ^f^	3.13 ± 0.13 ^de^	218.18 ± 8.62 ^cde^	76.48 ± 11.73 ^bcd^
Control	15	0	0	100	0.04 ± 0.01 ^a^	0.70 ± 0.04 ^a^	52.59 ± 3.74 ^a^	25.50 ± 2.80 ^a^

Data are mean ± standard deviation of three replicates. Different letters indicate statistically significant differences among different formulations for each biscuit type (ANOVA, Bonferroni *post hoc* test, *p* ≤ 0.05). Abbreviations: BQK, biscuits formulated with sprouted quinoa (X_1_), sprouted kiwicha (X_2_), and wheat flour (X_3_); BQC, biscuits formulated with sprouted quinoa (X_1_), sprouted cañihua (X_2_), and wheat flour (X_3_); BKC biscuits formulated with sprouted kiwicha (X_1_), sprouted cañihua (X_2_), and wheat flour (X_3_); GABA, γ-aminobutyric acid; GAE, gallic acid equivalents; ORAC, oxygen radical absorbance capacity; PA, phytic acid; TSPC, total soluble phenolic compounds; TE: Trolox equivalents.

**Table 3:** Predictive regression models describing the relationships between the nutritional and bioactive attributes of the biscuit with a mixed composition of flours.

Biscuit Type	Dependent Variables	Mathematical Models	R^2^ (Pred)	R^2^ (Adj)
BQK	PA	ŷ = 0.25X_1_ + 0.44X_2_ + 0.18X_3_	0.88	0.80
	GABA	ŷ = −39.67X_1_ − 41.12X_2_ − 31.30X_3_ + 179.69X_1_X_2_ + 154.65X_1_X_3_ + 161.99X_2_X_3_	0.76	0.71
	TSPC	ŷ = 130.89X_1_ + 291.14X_2_ + 368.00X_3_	0.78	0.74
BQC	TSPC	ŷ = −1.33X_1_ + 4.00X_2_ + 2.34X_3_ + 0.67X_1_X_2_	0.86	0.89
	ORAC	ŷ = 2.68X_1_ + 4.24X_2_ − 0.17X_3_	0.84	0.87
BKC	PA	ŷ = 0.25X_1_ + 0.18X_2_ + 0.10X_3_	0.80	0.81
	GABA	ŷ = 3.02X_1_ + 4.18X_2_ + 2.14X_3_	0.74	0.70
	ORAC	ŷ = 65.46X_1_ + 133.01X_2_ + 66.30X_3_	0.74	0.69

Abbreviations: BQK, biscuits formulated with sprouted quinoa (X_1_), sprouted kiwicha (X_2_), and wheat flour (X_3_); BQC, biscuits formulated with sprouted quinoa (X_1_), sprouted cañihua (X_2_), and wheat flour (X_3_); BKC biscuits formulated with sprouted kiwicha (X_1_), sprouted cañihua (X_2_), and wheat flour (X_3_); GABA, γ-aminobutyric acid; GAE, gallic acid equivalents; ORAC, oxygen radical absorbance capacity; PA, phytic acid; TSPC, total soluble phenolic compounds; TE: Trolox equivalents.

**Table 4:** Composition of flour blends and predicted values for PA, GABA, TSPCs, and ORAC in biscuits at optimum desirability value (D).

Biscuit Type	Optimum Desirability Value (D)	Flour Formulation at Optimum D	Response Variables	Predicted Values	−95% CI	+95% CI
BQK	0.487	21% SQF, 16% SKF, 63% WF	PA (g/100 g)	0.24	0.16	0.33
			GABA (mg/100 g)	13.57	2.44	17.78
			TSPC (mg GAE/100 g)	177.95	125.72	246.49
BQC	0.959	15% SQF, 25% SCF, 60% WF	TSPC (mg GAE/100 g)	472.44	437.96	506.92
			ORAC (μmol TE/g)	135.87	124.70	147.04
BKC	0.704	5% SKF, 23% SCF, 72% WF	PA (g/100 g)	0.15	0.11	0.19
			GABA (mg/100 g)	3.37	3.09	3.65
			ORAC (μmol TE/g)	106.53	95.38	117.67

Abbreviations: BQK, biscuits formulated with sprouted quinoa, sprouted kiwicha, and wheat flours; BQC, biscuits formulated with sprouted quinoa, sprouted cañihua, and wheat flours; BKC, biscuits formulated with sprouted kiwicha, sprouted cañihua, and wheat flours; CI: confidence interval; GABA, γ-aminobutyric acid; GAE, gallic acid equivalents; ORAC, oxygen radical absorbance capacity; PA, phytic acid; SCF: sprouted cañihua flour; SKF, sprouted kiwicha flour; SQF, sprouted quinoa flour; TSPC, total soluble phenolic compounds; TE: Trolox equivalents; WF: refined wheat flour.

**Table 5:** In vitro starch digestibility and changes in PA, GABA, TSPCs, and AA during different phases of digestion for control biscuit (100% WF) and BQC prepared with the optimal formulation (15% SQF, 25% SCF, and 60% WF).

Digestion Phase/Time	Parameters	Control Biscuit	BQC
None/0 min	PA (g/100 g)	0.21 ± 0.01 ^a^	0.25 ± 0.00 ^a,^*
	GABA (mg/100 g)	0.7 ± 0.04 ^a^	6.23 ± 0.06 ^b,^*
	TSPC (mg GAE/100 g)	52.59 ± 3.74 ^a^	428.78 ± 6.39 ^a,^*
	ORAC (μmol TE/g)	25.51 ± 2.80 ^a^	136.25 ± 2.19 ^b,^*
Gastric/120 min	PA (g/100 g)	0.32 ± 0.01 ^a^	0.25 ± 0.07 ^a,^*
	GABA (mg/100 g)	0.67 ± 0.06 ^a^	6.34 ± 0.48 ^a,^*
	TSPC (mg GAE/100 g)	128.29 ± 0.99 ^b^	429.28 ± 6.77 ^a,^*
	ORAC (μmol TE/g)	41.69 ± 0.62 ^b^	119.47 ± 12.60 ^ab,^*
Intestinal/120 min	PA (g/100 g)	0.26 ± 0.39 ^a^	0.39 ± 0.04 ^b,^*
	GABA (mg/100 g)	1.75 ± 0.06 ^b^	8.97 ± 0.11 ^b,^*
	TSPC (mg GAE/100 g)	401.73 ± 8.19 ^c^	638.49 ± 2.65 ^b,^*
	ORAC (μmol TE/g)	103.59 ± 8.02 ^c^	195.26 ± 8.48 ^c,^*
In vitro starch digestibility	HI	87.3 ± 0.27	75.99 ± 0.96 *
	AUC	27,633.1 ± 85.8	24,046.0 ± 304.5 *
	GI	87.65 ± 0.15	81.43 ± 0.53 *

Data are mean ± standard deviation of three replicates. * Denotes statistically significant differences between mean values of control and BQC data sets (Dunnett’s *post hoc* test, *p* ≤ 0.05). Different letters show statistical differences among mean values after different phases of digestion (ANOVA, Bonferroni *post hoc* test, *p* ≤ 0.05). Abbreviations: AUC, area under the curve; BQK, biscuits formulated with sprouted quinoa, sprouted kiwicha, and wheat flours; BQC, biscuits formulated with sprouted quinoa, sprouted cañihua, and wheat flours; GABA, γ-aminobutyric acid; GAE, gallic acid equivalents; GI: glycemic index; HI: hydrolysis index; ORAC, oxygen radical absorbance capacity; PA, phytic acid; SCF: sprouted cañihua flour; SKF, sprouted kiwicha flour; SQF, sprouted quinoa flour; TSPC, total soluble phenolic compounds; TE: Trolox equivalents; WF: refined wheat flour.

## Data Availability

The data presented in this study are available in this article or supplementary materials.

## Supplementary Materials

The following are available online at https://www.mdpi.com/article/10.3390/foods11111533/s1, Figure S1: Starch hydrolysis kinetic of control biscuit (100% WF), BQC (15% SQF, 25% SCF, and 60% WF) and white wheat bread, Table S1: Sprouting parameters of cañihua, kiwicha, and quinoa grains, Table S2: Experimental design with three independent variables (proportion of a combination of three flour types).

## Abbreviation

AUC: area under the curve; BQK, biscuits formulated with sprouted quinoa, sprouted kiwicha and wheat flours; BQC, biscuits formulated with sprouted quinoa, sprouted cañihua and wheat flours; BKC, biscuits formulated with sprouted kiwicha, sprouted cañihua and wheat flours; CF, cañihua flour; dw, dry weight; GABA, γ-aminobutyric acid; GAE, gallic acid equivalents; GI, glycemic index; HI, hydrolysis index; KF, kiwicha flour; ORAC, oxygen radical absorbance capacity; PA, phytic acid; QF, quinoa flour; SCF: sprouted cañihua flour; SKF, sprouted kiwicha flour; SQF, sprouted quinoa flour; TSPC, total soluble phenolic compounds; TE, Trolox equivalents; WF, refined wheat flour.
