# Supplementary material for: Improving Nutritional and Health Benefits of Biscuits by Optimizing Formulations Based on Sprouted Pseudocereal Grains
Source: Foods. 2022 May 24;11(11):1533. doi: 10.3390/foods11111533 (PMC9180627; doi:10.3390/foods11111533)
Supplement: Supplementary file 1 [file foods-11-01533-s001.zip › foods-1737615-supplementary.pdf]

foods

MDPI

Supplementary material

Improving Nutritional and Health Benefits of Biscuits by Optimizing Formulations Based on Sprouted Pseudocereal Grains from Andean Origin

Luz María Paucar-Menacho [1], Wilson Daniel Simpalo-López [1], Williams Estiward Castillo-Martínez [1], Lourdes Jossefyne Esquivel-Paredes [1] and Cristina Martínez-Villaluenga [2,*]

[1] Departamento de Agroindustria y Agronomía, Facultad de Ingeniería, Universidad del Santa, 02711 Nuevo Chimbote, Ancash, Perú; luzpaucar@uns.edu.pe (L.P.-M.); wsimpalol@uns.edu.pe (W.D.S.-L.); wcastillo@uns.edu.pe (W.E.C.-M.); lourdes.ep@gmail.com (L.J. E-P.)

[2] Department of Food Characterization, Quality and Safety, Institute of Food Science, Technology and Nutrition (ICTAN-CSIC), 28040 Madrid, Spain; c.m.villaluenga@csic.es (C. M-V.)

* Correspondence: c.m.villaluenga@csic.es (C. M-V.); Tel.: +34913939927

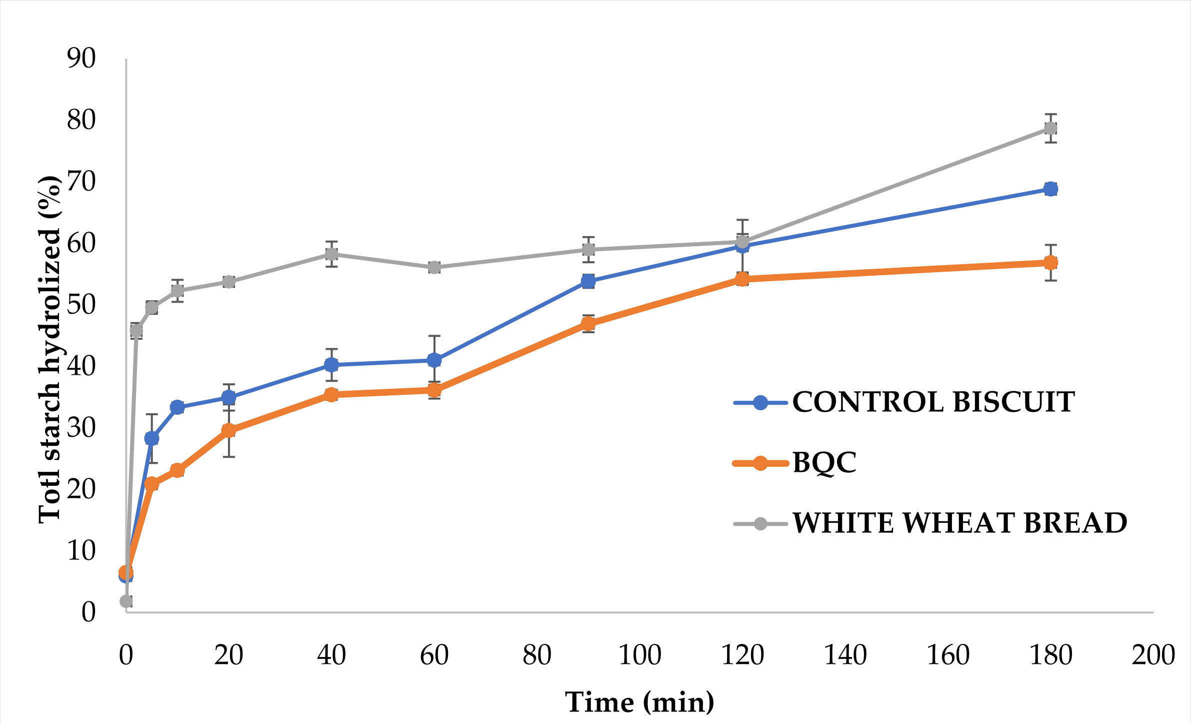

Figure S1. Starch hydrolysis kinetic of control biscuit (100% WF), BQC (15% SQF, 25% SCF, and 60% WF) and white wheat bread (100% WF).

Table S1. Sprouting conditions of cañihua, kiwicha, and quinoa grains.

Grains	Temperature (°C)	Time (h)	Germination Rate (%)
Cañihua	20	72	98
Kiwicha	26	63	100
Quinoa	20	42	95

The optimal germination temperature and time of cañihua, kiwicha and quinoa was established based on previous optimization studies [1-3].

Table S2. Experimental design with three independent variables (blends of three flour types).

Recipe no.	Proportion of Flours[a]		
	Sprouted Pseudocereal Flour 1 (X_1)	Sprouted Pseudocereal Flour 2 (X_2)	Wheat Flour (X_3)
1	15	15	70
2	20	20	60
3	5	20	75
4	20	20	60
5	5	20	75
6	5	20	75
7	20	20	60
8	10	25	65
9	20	5	75
10	20	5	75
11	10	10	80
12	25	10	65
13	20	5	75
14	25	10	65

[a] Composition of flour (expressed as %; sum of ingredients = 100%) used in elaboration of biscuit dough: BQK (X_1: sprouted quinoa flour; X_2: sprouted kiwicha flour; X_3: wheat flour), BQC (X_1: sprouted quinoa flour; X_2: sprouted cañihua flour; X_3: wheat flour) and BKC (X_1: sprouted kiwicha flour; X_2: sprouted cañihua flour; X_3: wheat flour).

Abreviations:

BQC: biscuit formulated with sprouted quinoa, sprouted cañihuaa and wheat flours; SCF: sprouted cañihua flour; SQF, sprouted quinoa flour; WF: re-fined wheat flour.

References

1. Paucar-Menacho, L.M.; Peñas, E.; Dueñas, M.; Frias, J.; Martínez-Villaluenga, C. Optimizing germination conditions to enhance the accumulation of bioactive compounds and the antioxidant activity of kiwicha (Amaranthus caudatus) using response surface methodology. *LWT-Food Sci. Technol.* **2017**, *76*, 245-252, doi:10.1016/j.lwt.2016.07.038.
2. Paucar-Menacho, L.M.; Martínez-Villaluenga, C.; Dueñas, M.; Frias, J.; Peñas, E. Response surface optimisation of germination conditions to improve the accumulation of bioactive compounds and the antioxidant activity in quinoa. *Int. J. Food Sci. Technol.* **2018**, *53*, 516-524, doi:10.1111/ijfs.13623.
3. Abderrahim, F.; Huanatico, E.; Repo-Carrasco-Valencia, R.; Arribas, S.M.; Gonzalez, M.C.; Condezo-Hoyos, L. Effect of germination on total phenolic compounds, total antioxidant capacity, Maillard reaction products and oxidative stress markers in canihua (Chenopodium pallidicaule). *J. Cereal Sci.* **2012**, *56*, 410-417, doi:https://doi.org/10.1016/j.jcs.2012.04.013.
